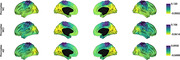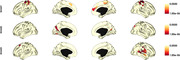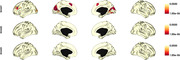# Gradient Analysis for the Study of Alzheimer’s Disease

**DOI:** 10.1002/alz.093615

**Published:** 2025-01-09

**Authors:** Aldo Camargo, Ze Wang

**Affiliations:** ^1^ University of Maryland of Baltimore, Baltimore, MD USA

## Abstract

**Background:**

Dys‐connectivity has been repeatedly shown in Alzheimer’s Disease (AD) but the change of connectivity gradient across the brain is under‐studied. In this study, we used resting state fMRI (rsfMRI) from the Alzheimer’s Disease Neuroimaging Initiative (ADNI) to build a whole brain functional connectivity matrix. We then compared the major connectivity gradients decomposed from the connectivity matrix from normal controls (NC), mild cognitive impairment (MCI), and AD patients.

**Method:**

30 NC, 11 MCI, and 40 AD were included in the analysis. Data preprocessing including motion correction, slice time correction, normalization, and artifact component removal, was performed using SPM12 and FSL. Mean rsfMRI time series was extracted from each of the 400 segments in the Schaefer atlas. A 400×400 functional connectivity matrix was calculated for each subject. Connectivity gradients were subsequently calculated using the BrainSpace toolbox. A mean connectivity matrix and its gradients were calculated for NC, MCI, and AD separately. Individual subject’s gradients were then aligned to those population level ones and compared across groups through two‐sample t‐test. Our analysis limits to the first three gradients as the corresponding eigenvalues explained >39% of the original matrix variance. Multiple comparison correction was performed using Bonferroni correction.

**Result:**

Fig. 1 shows the first gradient map for each sub‐group. The gradients were lowest in occipital cortex, and increased in the motor network and temporal cortex, and then gradually went down toward prefrontal cortex. Fig. 2‐4 are the statistical comparison results. The first gradient map showed the largest cross‐sectional changes. Compared to NC, MCI had reduced gradients in occipital cortex, motor cortex, and temporal cortex. Gradients in occipital cortex and posterior part of temporal cortex further reduced in AD compared to MCI, and AD patient showed gradient reduction in prefrontal cortex.

**Conclusion:**

We found consistent functional connectivity gradient reduction in the AD continuum in occipital cortex and temporal cortex, suggesting a common pathway of AD‐related impairment to functional connectivity. Gradient reduction was identified in motor cortex in MCI compared to NC and was found in prefrontal cortex in AD compared to MCI, likely related to the escalated cognitive control and working memory decline in AD.